# Multi-Approach Bioinformatics Analysis of Curated Omics Data Provides a Gene Expression Panorama for Multiple Cancer Types

**DOI:** 10.3389/fgene.2020.586602

**Published:** 2020-11-23

**Authors:** Bruno César Feltes, Joice de Faria Poloni, Itamar José Guimarães Nunes, Sara Socorro Faria, Marcio Dorn

**Affiliations:** ^1^Laboratory of Structural Bioinformatics and Computational Biology, Institute of Informatics, Federal University of Rio Grande do Sul, Porto Alegre, Brazil; ^2^Center of Biotechnology, Federal University of Rio Grande do Sul, Porto Alegre, Brazil; ^3^Laboratory of Immunology and Inflammation, Department of Cell Biology, University of Brasilia, Brasilia, Brazil; ^4^National Institute of Science and Technology - Forensic Science, Porto Alegre, Brazil

**Keywords:** regulatory networks, overall survival, machine learning, omics, bioinformatics, systems biology, cancer

## Abstract

Studies describing the expression patterns and biomarkers for the tumoral process increase in number every year. The availability of new datasets, although essential, also creates a confusing landscape where common or critical mechanisms are obscured amidst the divergent and heterogeneous nature of such results. In this work, we manually curated the Gene Expression Omnibus using rigorous filtering criteria to select the most homogeneous and highest quality microarray and RNA-seq datasets from multiple types of cancer. By applying systems biology approaches, combined with machine learning analysis, we investigated possible frequently deregulated molecular mechanisms underlying the tumoral process. Our multi-approach analysis of 99 curated datasets, composed of 5,406 samples, revealed 47 differentially expressed genes in all analyzed cancer types, which were all in agreement with the validation using TCGA data. Results suggest that the tumoral process is more related to the overexpression of core deregulated machinery than the underexpression of a given gene set. Additionally, we identified gene expression similarities between different cancer types not described before and performed an overall survival analysis using 20 cancer types. Finally, we were able to suggest a core regulatory mechanism that could be frequently deregulated.

## 1. Introduction

Despite the breakthroughs made every year, cancer is still the second leading cause of death worldwide (Bray et al., 2018), and continuous efforts must be made to understand the molecular mechanisms underlying this disease and engage new future treatment options. Taking a step back from a preventive (i.e., personal screening) point of view, one of the significant issues to understanding cancer biology is its inherent heterogeneous nature, as has been seen in various cancer types (Shen et al., 2016; Hardiman, 2018; Ho et al., 2018; Joseph et al., 2018; Zhang et al., 2018). It thus becomes difficult to identify the primary molecular drivers of the tumoral process and to understand the differences between distinct cancer types without comparing their expression patterns to examine their most prominent variations. Nowadays, there are multiple sources of gene expression data, such as microarray and RNA-seq analysis, both of which provide valuable information that can be employed to create a more accurate portrait of these molecular differences. Due to the massive amount of works published each year identifying tumoral expression patterns and biomarkers, however, that could be biologically relevant in those contexts it is neither intuitive nor clear. Consequentially, the most optimal approach to create this expression panorama would be to combine both types of data and design a multi-approach computational strategy to extract relevant information, as different computational strategies provide distinct results and interpretations. The use of multiple types of data and different computational approaches to understand the molecular mechanisms underlying the tumoral process and comprehend what could lie beyond the heterogeneity in cancer is already broadly accepted (Archer et al., 2016; Doherty et al., 2019; Olivier et al., 2019). Additionally, a distinct dataset-mining protocol would be fundamental to assure proper comparison and valid results, which is a subject that, unfortunately, is not adequately discussed and frequently overlooked in most computational research.

Nowadays, pan-cancer studies are among the promising approaches to find new genes that can have a potential role as prognosis biomarkers or that have therapeutic uses. There are numerous alternatives when conducting a pan-cancer study. For example, they can follow a pure machine-learning approach, exploring the combination of omics-data (Gonzalez-Reymundez and Vazquez, 2020), the combination of different types of cancer data (Chiu et al., 2019), or applying diverse protocols to answer questions on specific data types, such as immunological (Polano et al., 2019), mutations (Palazzo et al., 2019), methylation (Yang X. et al., 2017; Saghafinia et al., 2018), proteomics (Akbani et al., 2014), lncRNA (Li Y. et al., 2018), miRNA (Cheerla and Gevaert, 2017), or mRNA data (Demircioğlu et al., 2019). Likewise, they can also be accompanied by network approaches (Chen et al., 2018; Cava and Castiglioni, 2019), signaling pathway analysis (Neapolitan et al., 2015), or networks associated with functional pathways analysis (Cava et al., 2018). Pan-cancer studies are conducted using various methods, all of which are valid in their way and adapted to their own objectives. Nevertheless, in this pursuance of mass analyzing cancer datasets, creating new, rigorous, and integrative approaches is fundamental (Doherty et al., 2019) to maintaining the stimulation of further discussions and devising new analytical choices.

In this work, we provide a multi-approach bioinformatic study of both microarray and RNA-seq data, combined this with a machine learning (ML) approach and systems biology tools to access the main expression similarities and differences of various cancer types. By manually curating the Gene Expression Omnibus (GEO) [ncbi.nlm.nih.gov/geo/], and individually examining the available cancer-related microarray and RNA-seq experiments up to 2018, we gathered only the most reliable and homogeneous datasets for further analysis. From 82 microarray experiments and 17 RNA-seq datasets, we were able to devise a panorama explaining the distinct gene expression patterns found in multiple tumoral types. Additionally, we compared the classical approach of expression analysis with an ML approach with 4,074 pooled samples to observe the specific tumoral signatures obtained in both cases. Our multi-approach revealed a possible frequently deregulated machinery in common between all analyzed cancer types that was supported by multiple approaches and validated using TCGA data. We have assessed a possible prevalent molecular pathway that could be responsible for the tumoral process, and we have provided an overall survival analysis for additional discussion. We have highlighted that this work aims to create and report results of a new, rigorous, and reproducible multi-approach *in silico* approach. Comparing different methods from the heterogeneous protocols employed by pan-cancer studies falls beyond the scope of this work.

## 2. Materials and Methods

### 2.1. Omics Data Obtainment

To obtain multiple omics datasets (GSEs) of microarray and RNA-seq data, we manually mined all GSEs currently available at the GEO database related to colorectal, gastric, bone, skin, pancreatic, liver, bladder, lung, head/neck, renal, brain, prostate, uterine, ovarian, and breast cancers. We also included thyroid and parathyroid cancers within the head/neck category. The criteria applied to select the most reliable and homogeneous datasets were as follows: (i) exclusion of studies that employed any type of pharmacological manipulation; (ii) exclusion of studies that used interfering molecules, such as miRNAs, siRNAs, or used gene therapies of any kind; (iii) elimination of datasets that applied knockdown cultures or artificially induced mutations; (iv) selection of studies with at least three control and three experimental samples; (v) selection of studies performed exclusively in *Homo sapiens*; (vi) removal of studies that used xenograft techniques; (vii) selection of studies that only presented a clear description of protocol or samples employed (i.e., at least correctly labeled); (viii) selection of datasets that made their raw data available, thus excluding all datasets that only made accessible author's treated data; (ix) removal of studies performed in platforms not belonging to Affymetrix, Illumina, or Agilent manufactures; (x) exclusion of samples in metastasized tissues; and (xi) exclusion of leukemia studies. The same rigorous filtering criteria were applied for microarray and RNA-seq datasets. In the end, all studies up to the end of 2018 were individually examined and manually curated. A total of 82 microarray datasets (including both single and dual channel experiments) and 17 RNA-seq studies were selected for further analysis. A similar search protocol was employed to construct our curated microarray database for machine learning benchmarking and testing, named Curated Microarray Database (CuMiDa) [http://sbcb.inf.ufrgs.br/cumida] (Feltes et al., 2019).

### 2.2. Preprocessing and Differential Gene Expression Analysis of Microarray Data

The *GEOquery* package (Davis and Meltzer, 2007) for the R platform was employed to download the raw data of the selected microarray studies. If any samples displayed errors or file corruption during preprocessing they were manually excluded. Each of the 82 dataset were individually submitted to background correction and normalization. In this sense, the following packages were employed: (i) *affy* (Gautier et al., 2004) for Affymetrix-derived datasets; (ii) *lumi* (Du et al., 2008), *beadarray* (Dunning et al., 2007), and *illuminaio* (Smith et al., 2013) for Illumina-derived datasets; and (iii) package *limma* (Ritchie et al., 2015) for Agilent and other platforms. After preprocessing, all microarrays datasets were analyzed by the *arrayQualityMetrics* (Kauffmann et al., 2009) package to access the sample quality information. In all cases, samples that displayed a low quality in at least half of the parameters measured by *arrayQualityMetrics* were excluded prior to differential gene expression analysis. Finally, the packages *limma* and *Biobase* (Huber et al., 2015) were employed during differential gene expression analysis. Differentially Expressed Genes (DEGs) were obtained by applying a filter of |*log*2*FC*|≥1 with the Benjamini-Hochberg for FDR correction of *p* < 0.05. Datasets were individually analyzed. Jaccard indexes were accessed using the GeneOverlap R package (Shen and Sinai, 2020).

### 2.3. Preprocessing and Differential Gene Expression Analysis of RNA-Seq Data

The raw data of the 17 previously selected datasets were submitted for quality analysis using FastQC application [http://www.bioinformatics.babraham.ac.uk/projects/fastqc], and this was followed by the trimming of low-quality bases, poly-N sequences, remaining ribosomal RNA, and adapter sequences using the Trimmomatic 0.35 software (Bolger et al., 2014). The resulting data were mapped against the reference genome of *Homo sapiens* (Ensembl version GRCh38.94) using the software STAR v2.6.0a in combination with RSEM v1.3.1 to achieve the transcript abundance quantification (Li and Dewey, 2011; Dobin et al., 2013). To estimate the differential gene expression, the transcript quantification resulting from RSEM was used as input in the tximport and DESeq2 R packages (Love et al., 2014; Soneson et al., 2015). Differential gene expression was determined by considering FDR *p* < 0.05 and |*log*2*FC*| ≥ 1. Datasets were individually analyzed. Jaccard indexes were accessed using the GeneOverlap R package (Shen and Sinai, 2020).

### 2.4. Machine Learning Approach

To identify possible gene expression patterns in the chosen datasets, we used our previously described neuroevolution-based microarray analysis tool, N3O (Grisci et al., 2019). In short, N3O uses the Feature Selection-Neuroevolution of Augmenting Topologies (FS-NEAT) as the main algorithm (Miao and Niu, 2016), but it was adapted for high-dimensional data using new structural operators. Additionally, N3O avoids overfitting by using a modified L2 regularization in its fitness function and by performing feature selection. For more on how N3O was adapted deal with microarray data, please see Grisci et al. (2019). N3O was created to simultaneously classify microarray data and select the subset of more relevant genes. We used the data available in the CuMiDa database as input for N3O since all available gene expression matrices in CuMiDa were already previously curated and adapted to machine learning protocols—the 78 datasets in CuMiDa are derived from the 82 datasets employed in this work, but CuMiDa also has leukemia datasets, which were omitted (Feltes et al., 2019). The reason to omit leukemia datasets is explained further. As stated in the CuMiDa publication, many classes (conditions) needed to be excluded because they did not fit the minimum requirement for an ML protocol. This happened because the success of an ML approach is directly linked to a minimum and maximum amount of samples. The datasets in CuMiDa have a minimum amount of six samples per condition because fewer samples would severely impact the performance of any ML algorithm. Any condition with fewer than six samples therefore needed to be excluded prior to the statistical treatment, as stated in the CuMiDa publication. Some classes are consequently missing and were thus not taken into consideration in the comparisons described in the next sections. Moreover, all samples from the microarray analysis were pooled together, by tissue type, separating normal tissues from tumoral tissues, to be further analyzed together by N3O. Each pool was batch effect corrected using *limma*. This analysis was performed in parallel with the previous ones. Additionally, to further deal with class imbalance, N3O uses an altered binary cross-entropy function to compute the fitness of the neural network. The cross-entropy is computed individually for each class and then averaged; all classes therefore have the same contribution to the fitness, independently of their sizes (Grisci et al., 2019).

Input matrices were adapted before serving as input on N3O because of the inherited imbalance number of probes provided by each platform. Since multiple probes can point to a single gene and vice-versa, probes were chosen based on the following criteria: (i) when multiple probes were assigned to the same gene, the more specific ones (i.e., assigned to a single gene) were prioritized; (ii) if more than one probe was particularly assigned to one gene, the first occurrence was taken into consideration, thus deleting the remaining duplicates (in this case, we manually checked the probes in case the expression values were significantly different before excluding them); and (iii) to merge multiple platforms, the gene IDs that were not intersected between the matrices were removed.

### 2.5. Systems Biology Analysis

To further analyze the major molecular pathways related to the final selected targets, we conducted a systems biology approach. To generate the primary protein-protein interaction (PPI) networks, we employed the STRING 11 metasearch engine (Szklarczyk et al., 2018). All targets, which will be mentioned further, were simultaneously searched, and the networks were saturated until all inputted proteins were connected (or almost connected). The parameters in STRING were as follows: (i) degree of confidence of 0.400; (ii) only experiments and co-expression search parameters enabled; and (iii) no more than 200 interactors on the first shell and no interactors on the second shell. Afterward, networks were imported into the Cytoscape 3.7.1 software for analysis and manipulation (Shannon et al., 2003). The topology of each network was analyzed in terms of clustering, centralities, and gene ontology, aiming to find the most topologically relevant nodes and molecular pathways related to each PPI network. The Cytoscape plug-in CentiScaPe 2.2 (Scardoni et al., 2009) was employed to analyze the following centralities: node degree, which calculates the number of immediate connections of a given node; betweenness, which calculates the number of shortest paths that pass through each node; and eigenvector, a centrality measure that calculates how regulatory a node is based on the node's number of connections and how well connected their neighbors are. Possible signaling pathways were calculated using PathLinker (Huang et al., 2018). The parameters in Pathlinker were as follows: (i) k: 500; (ii) edge penalty: 1; (iii) edge weight: unweighted; (iv) treat network as undirected option disabled; (v) allow sources and targets in path option enabled; and (vi) connect sources to each other option enabled.

### 2.6. Functional Enrichment and Transcription Factor Prospection

For functional enrichment analysis, ClueGO 2.5.5 was employed (Bindea et al., 2009). In ClueGO, the two-sided hypergeometric test was used in combination with the Bonferroni family-wise test with a significance of *p* < 0.05. Additional parameters included the following: (i) GO evidence, all experimental; (ii) Ontologies/Pathways, GO biological processes from UniProt; and (iii) Selected Ontologies Reference Set enabled. Processes under x10^−^5, after the Bonferroni test, were not taken into consideration. General processes that did not represent informative results (e.g., regulation of the biological process, metabolism, positive/negative regulation of biological processes, etc.) were also excluded. To further select the most representative bioprocesses, we focused on a more accurate description of a given biological activity, such as processes that indicated a positive or negative regulation. When both “negative” and “positive” regulation appeared in the GO list, we selected the one with the highest significance. Finally, we only took into consideration processes that appeared at least three times for each category (e.g., DNA repair, cell cycle, etc.); processes with fewer than three were judged as artifacts. Finally, the TRRUST v2 (Han et al., 2017) database was used for prospecting the transcription factors (TF) associated with the top regulatory genes from the predicted signaling pathways analyzed by the previous Systems Biology approach.

### 2.7. Overall Survival Analysis and Gene Expression Validation

The overall survival analysis was performed using the Gene Expression Profiling Interactive Analysis 2 (GEPIA 2) [http://gepia.cancer-pku.cn/index.html] tool (Tang et al., 2017). The data employed by GEPIA originates from the Cancer Genome Atlas (TCGA) and Genotype-Tissue Expression (GTEx) project. Survival analysis based on gene expression levels was conducted by using the log-rank test, represented in the form of Kaplan-Meier plots. For the analysis, a *p* < 0.05 was considered, together with the cox proportional hazard ratio and 95% confidence interval information.

The analyzed genes were divided into two classes based on the quartile expression. Patients above the upper quartile were classed as the high expression group, and those below were classed as the low expression group.

We performed pan-cancer screening for survival in 20 cancer subtypes: (i) bladder carcinoma (BLCA); (ii) breast invasive carcinoma (BRCA); (iii) colon adenocarcinoma (COAD); (iv) glioblastoma multiforme (GBM); (v) low grade glioma (LGG); (vi) head-neck squamous cell carcinoma (HNSC); (vii) kidney renal clear cell carcinoma (KIRC); (viii) kidney renal papillary cancer (KIRP); (ix) Chromophobe renal cancer (KICH); (x) lung adenocarcinoma (LUAD); (xi) lung squamous cell carcinoma (LUSC); (xii) ovarian serous cystadenocarcinoma (OV); (xiii) rectum adenocarcinoma (READ); (xiv) prostate adenocarcinoma (PRAD); (xv) thyroid carcinoma (THCA); (xvi) esophageal carcinoma (ESCA); (xvii) liver hepatocellular carcinoma (LIHC); (xviii) stomach adenocarcinoma (STAD); (xix) pancreatic adenocarcinoma (PAAD); and (xx) skin cutaneous melanoma (SKCM).

Furthermore, we used DriverDBv3, which employs data from TCGA, to validate our gene expression results (Liu et al., 2020).

## 3. Results and Discussion

### 3.1. Data Gathering, Differential Expression, and Feature Selection

It is common for omics-based works to download several expression datasets and analyze them using different methods, by either employing already known tools or developing newer ones. However, little is discussed regarding the quality of the downloaded datasets or which datasets were combined. The reality we face is that this approach combines several types of experiments, composed of different experimental protocols, creating an even more heterogeneous analytic environment for a disease that is already heterogeneous by nature. Even the broadest analysis, with thousands of samples, can consequently display compromised results.

To partially overcome such bias, we manually curated the entire GEO database for microarray and RNA-seq studies from the oldest of each type, up to the end of 2018, using rigorous filtering criteria. By excluding the most critical causes of deviation, we aimed to amass the most homogeneous pack of expression studies we could. The heterogeneous nature of cancer will always be a biological variable by itself, however, and the fact that all studies are performed by different groups is an intrinsic divergence as well. We also excluded leukemia works, thus focusing only on solid tumors. Taking this into consideration, we rationalized that comparing results from leukemia datasets with those from solid tumors would not be feasible. We considered thyroid cancer as part of the head/neck category since it is often treated as part of this class in the medical field and in the scientific literature (Arboleda et al., 2020). No cancer derived from skin and uterine cancers fitted the entire filtering criteria (i.e., from dataset filtering to preprocessing steps) for microarray datasets, whereas no datasets for bone, bladder, pancreatic, brain, gastric, and ovarian cancers could be selected for RNA-seq. Only one study for RNA-seq (GSE88741) was performed exclusively in cell lineages. Since this dataset was the only skin cancer dataset to pass our quality filtering criteria, we maintained it to have at least one representative of this cancer type. In the end, more than 30,000 studies were manually curated.

In the end, 82 microarray studies and 17 RNA-seq datasets fitted all required criteria. The initial list was longer, with more than 300 studies for microarray and 30 for RNA-seq. However, in the course of the analysis, many displayed corrupted samples, did not pass with the minimum amount of required samples (or reads) after the quality analysis, or, as is the case of RNA-seq, did not pass our read cut-off amount. Supplementary Tables 1, 2 lists all microarrays and RNA-seq datasets gathered in this work and their information, respectively. We treated tumor samples as one group and normal samples as another for DEG analysis for each dataset. We chose this approach because the motivation behind our study is to find global differences or similarities rather than timely or specific gene expression between cancer subtypes. After the expression analysis, we had a total of 15,944 overexpressed and 16,045 underexpressed DEGs among all microarrays and 24,047 overexpressed and 15,328 underexpressed DEGs from the RNA-seq analysis. To avoid sacrificing the discovery of new potential frequently expressed DEGs a |*log*2*FC*| ≥ 1 was chosen.

Additionally, to not solely rely on the classical DEG approach, we employed an ML technique to select relevant features from microarray studies. N3O was previously described in its related publication (Grisci et al., 2019) and displayed high accuracy values when compared to other gold-standard ML approaches. The idea of applying N3O was not only to combine a different strategy to our analysis protocol of DEGs analysis but also to observe possible convergences of selected features vs. obtained DEGs. Gene expression data is composed by thousands of genes (features) and a small number of samples, which can lead to the so called “curse of dimensionality” problem, where the model can easily overfit, which increases memory consumption, processing time, and diminish interpretability (Verleysen and François, 2005). To partially overcome such obstacle, we took several careful steps in treating our input. In this sense, we initially downloaded all data available in the CuMiDa database (Feltes et al., 2019), our previously described database. CuMiDa was based on the same datasets employed in this work but processed to be exclusively used in ML protocols. Proper dataset handling to be used as input for machine learning approaches were currently discussed as one of the challenges and limitations to the application of this approach in cancer research (Troyanskaya et al., 2020). This further supports the employment of both the CuMiDa database, which is composed of curated and newer microarray data, and N3O, which was trained and tested using these datasets.

Moreover, some datasets in the CuMiDa database were redundant, meaning they were performed in more than one platform. For those, we chose the platform with the highest amount of DEG in the expression analyzes to carry out the ML approach. This happened for GSE14520(U133B), GSE6919(U95B and C), and GSE 6344(U133B). We also excluded the leukemia datasets, and the dual-channel datasets (GSE62043, GSE8511, GSE38241, GSE60329, and GSE22804) because they do not make a distinction between normal and tumoral samples, which cannot be used by supervised algorithms like the one employed by N3O. Afterwards, we had to balance our features so they could be properly analyzed considering that supervised learning algorithms, such as the one employed by N3O, cannot analyze an unfixed number of rows. This lack of balance is an inherent problem in the field because different manufactures provide a variable number of probes, which can also vary by platforms of the same manufacture. Microarray tables were therefore merged based on the Entrez Gene ID related to each probe, as described in section 2. More information about the final input matrices can be found in Supplementary Table 15.

All samples were pooled together, divided by healthy and tumoral tissues, for each type of cancer, generating a total of 4,074 samples. This resulted in 12 different matrices, which were individually analyzed. A total of 2,281 unique features (genes) were obtained for all cancer. N3O was developed exclusively for microarray data; it was thus not applied for the RNA-seq datasets. The RNA-seq datasets were not analyzed by the ML approach due to the low number of samples in the final selected datasets, which could compromise the final results in a ML protocol.

**Figure 1:**
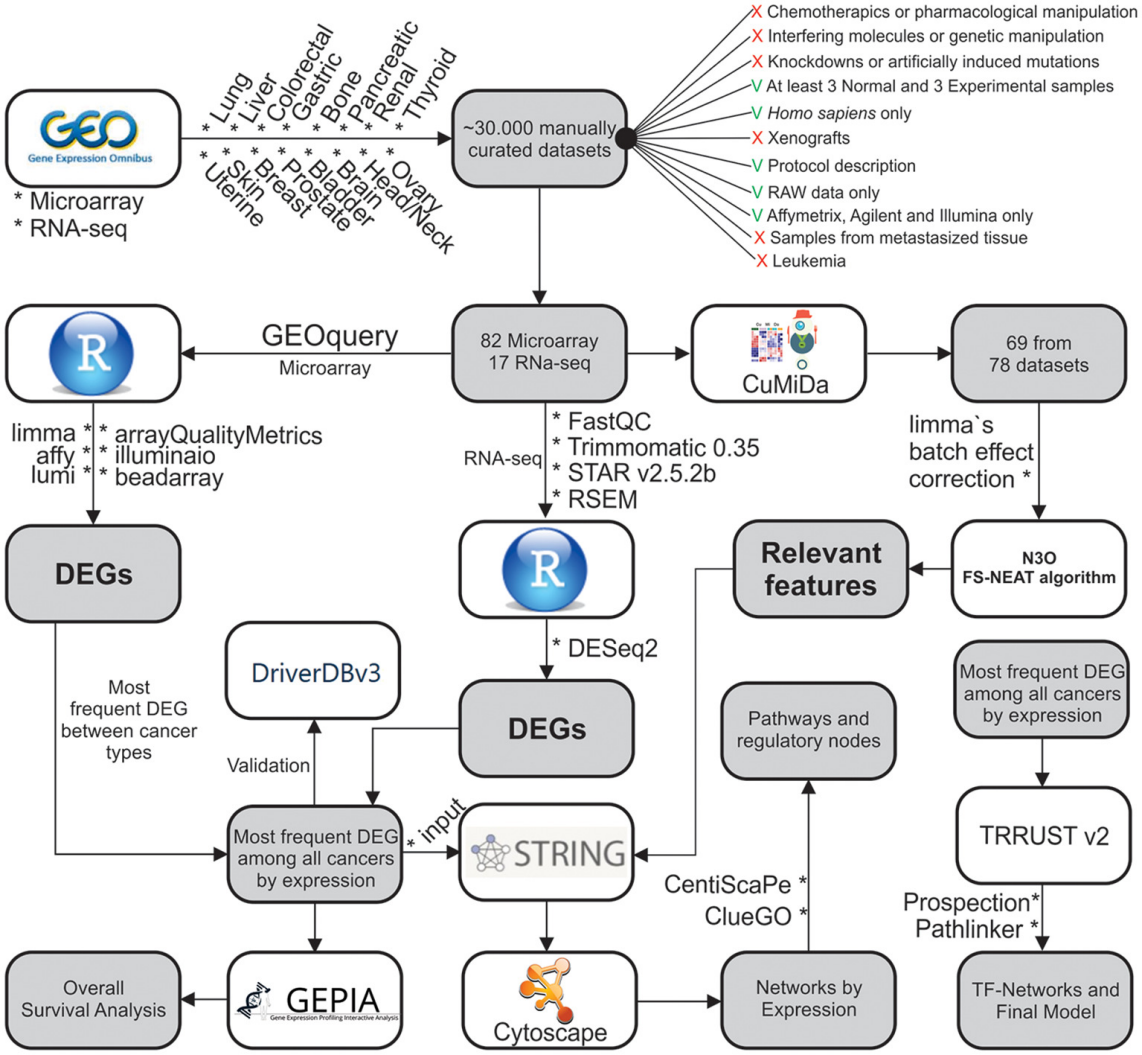
Methodological steps used in this work. The work is divided into: (1) Data gathering and curation; (2) Microarray analysis; (3) ML analysis of microarray data; (4) RNA-seq analysis; (5) Systems Biology approach; and (6) Overall Survival analysis.

The combination of these results was employed for the future analyzes described below. A summary of our methodological steps can be found on Figure 1.

### 3.2. Gene Expression Panorama of Multiple Cancer Types

Next, we sought to investigate the expression patterns underlying the DEG obtained from the previous analyzes. For the microarray results, overexpressed DEGs were matched between all GSEs from all cancer types and analyzed in terms of (i) quantity of most frequently expressed genes between cancer types (Supplementary Tables 3, 4); (ii) Jaccard index for the most frequently expressed genes between cancer types (Supplementary Table 5). The same analyses were performed for underexpressed genes (Supplementary Tables 6–8) and, for DEG obtained from the RNA-seq analysis, over- (Supplementary Tables 9–11) and underexpressed genes(Supplementary Tables 12–14). All tables are in Supplementary Material 2.

We first sought to determine which expression profiles were similar between each cancer type. Since it is not reasonable to assume that whole expression profiles could be identical due to the highly heterogeneous nature of cancer, we believed that Jaccard index values of 15% or higher were compelling observations. In this case, microarray results were separated from RNA-seq because RNA-seq studies tend to provide more extensive DEG lists, when compared to microarrays, deviating Jaccard index results.

**Figure 2:**
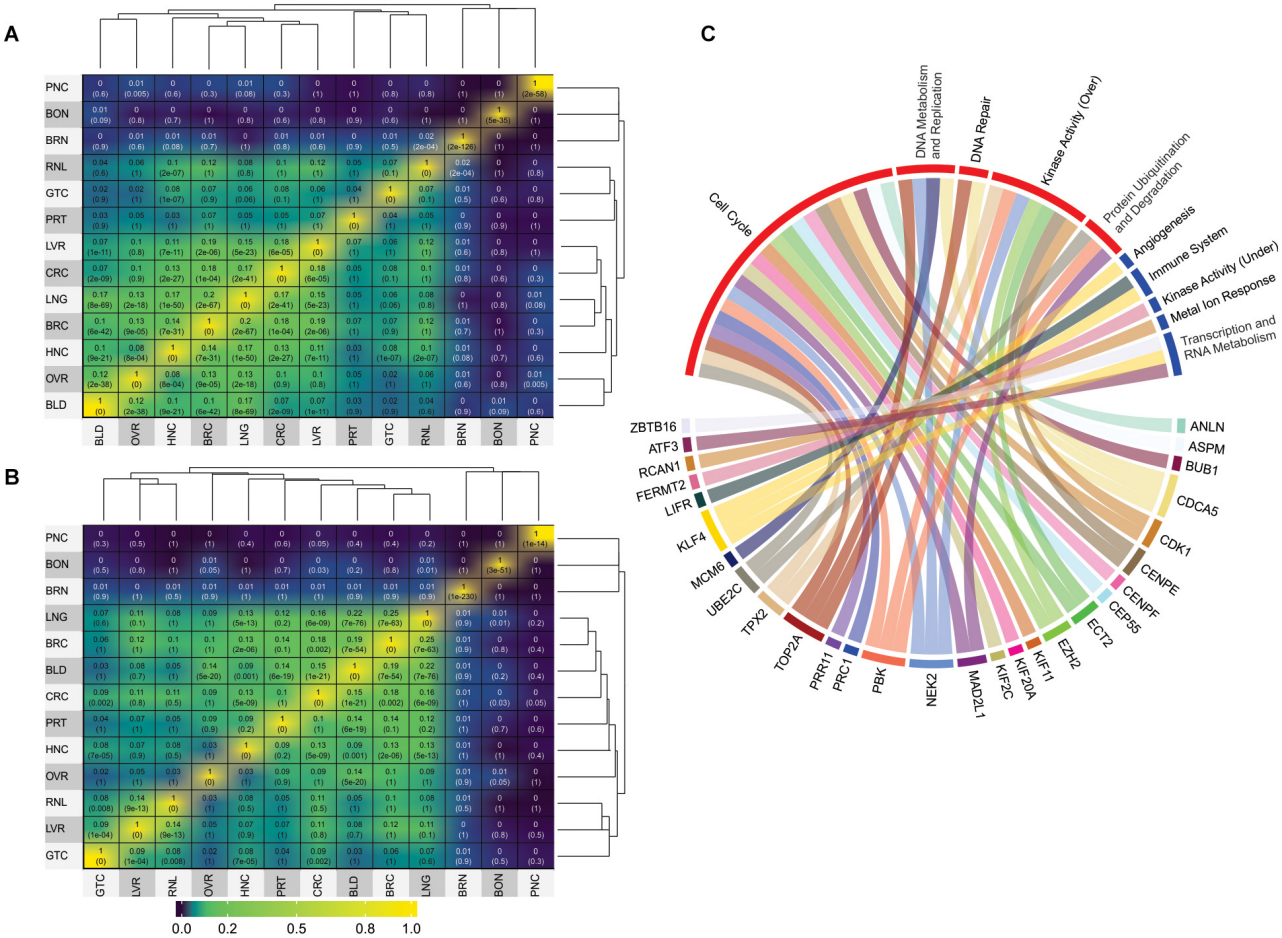
Jaccard index values and GO analysis. **(A)** Jaccard index matrix showing the similarity values for the overexpressed genes derived from the microarray analysis. **(B)** Jaccard index matrix showing the similarity values for the underexpressed genes derived from the microarray analysis. White labels were added just for clarity. RNA-seq matrices were not shows because they displayed no significant values based on our cut-off of at least 15% similarity; they can be found on Supplementary Material 2. The values can also be seen in Supplementary Tables 5, 8, 11, 14. PNC, pancreatic cancer; BON, bone [cancer]; BRN, brain [cancer]; RNL, renal [cancer]; GTC, gastric cancer; PRT, prostate [cancer]; LVR, liver [cancer]; CRC, colorectal cancer; LNG, lung [cancer]; BRC, breast cancer; HNC, head/Neck cancer; OVR, ovarian [cancer]; BLD, bladder [cancer]. **(C)** Gene Ontology groups for the 47 DEG frequently expressed between all analyzed cancer types. Details of which GO composed each group can be found in Supplementary Tables 16, 17.

Although it is expected that cancers in which we obtained a higher pool of datasets would present a higher similarity, such as breast, lung, liver, and colon cancer DEG expression profiles, some profile similarities were not anticipated. For example, overexpressed genes of bone, gastric, pancreatic, brain, and prostate cancers showed no significant similarity values to other types (Figure 2A). Although this was expected for bone since it has only one dataset, as well for pancreatic cancer, which has only two, prostate and gastric cancers were among the richest dataset groups (Supplementary Tables 1, 5). Prostate cancer might not have achieved our 15% similarity cut-off, but it came close, reaching 14% for breast and lung cancer for the underexpressed genes.

In contrast, one interesting highlight was the 16% similarity between overexpressed genes in head/neck and lung cancers (Figure 2A and Supplementary Table 5). Since head/neck were not among the richest in terms of dataset pool, whereas lung was, it is interesting that they have such a high similarity index. This result is in agreement with previous observations. By examining 3,527 tumoral samples of different cancer types, using both DNA and RNA data, a study published in 2014 was able to find significant correlations between head/neck and lung cancer, which they concluded to be more prone to similar treatments (Hoadley et al., 2014). A significant relationship between head/neck and lung cancer was also noted in a recent genome-wide based study that analyzed six cancer types from approximately 600,000 individuals. Amos et al. (2017), which further supports our results.

When we evaluated the data from underexpressed genes from microarray analyzes, the most salient results refers to the high similarity achieved by bladder cancer when compared to the other types. Bladder achieved similarity with breast (19%), colorectal (15%), lung (21%), and ovarian (19%) cancers (Figure 2B). Once again, although this is expected for groups with a rich dataset pool, it is unusual for bladder cancer, which has only two datasets. Although the relationship between, lung, breast, colorectal and ovarian cancers were observed in previous studies (Amos et al., 2017), no correlation was made so far with bladder cancer, making this a novel result. Yet, in general, overexpressed DEG showed more significant similarity indexes than underexpressed DEG.

Unfortunately, due to the low dataset pool achieved for RNA-seq, there were no significant similarities between the analyzed cancer types (Supplementary Figure 2). However, there were 33 overexpressed and 14 underexpressed genes found to be frequently deferentially expressed in all cancer types analyzed in this work (Table 1). Please note that these genes were not expressed in every single dataset, though they were those amidst the most frequently expressed. DEG should appear in at least six cancer types to be considered frequent for microarray analysis since it had more datasets (total of 82 datasets and 13 cancer types) and should appear at least three times for RNA-seq datasets (total of 17 datasets and eight cancer types). It is not feasible to assume that a given set of genes will be deregulated in all existing cancer types from tissues derived from distinct patients. Identifying the most frequently deregulated DEG, however, is a more realistic approach to understanding the complex molecular conundrum that is cancer.

**Table 1:** Table listing the 47 most frequently expressed DEG found between all analyzed cancer types, and their topological properties in the PPI-networks, when applied.

**Gene**	**Expression**	**General function**	**Topology**
ANLN	Over	Actin-binding protein related to citokinesis and migration.	HBS
ASPM	Over	Related to the mitotic spindle regulation.	HBS
ATAD2	Over	ATPase related to multiple cellular functions, including activation of oncogenes, like MYC.	HS
ATF3	Under	Member of the cAMP responsive element-binding factors	HBS
BIRC5	Over	Involved in the inhibition of apoptosis.	HBS
BUB1	Over	Plays a role in mitotic spindle-assembly, including the localization of CENPF and CENPE.	HBS
BUB1B	Over	Plays a role in mitotic spindle assembly and the localization of CENPE.	HBS
CDC25A	Over	Phosphatase involved in cell cycle progression.	S
CDCA5	Over	Protein associated with mitosis.	HS
CDK1	Over	Cyclin-Dependent Kinase deeply involved in cell cycle.	HBS
CENPE	Over	Member of the centromere-kinetochore complex.	HS
CENPF	Over	See CENPE.	HS
CEP55	Over	Centrosomal protein, associated to cytokinesis.	HBS
CTHRC1	Over	Putative roles in the negative regulation of collagen deposition.	NA
CYBRD1	Under	Member of the cytochrome b family, related to iron absorption.	B
DEPDC1	Over	Transcription corepressor, associated to apoptosis suppression and proliferation.	HS
ECT2	Over	Catalyzes the GDP-GTP exchange. Also involved in cytokenesis.	HS
EZH2	Over	Polycomb-group family, involved in gene silencing and DNA methylation.	B
FERMT2	Under	Involved in extracellular matrix adhesion and regulates cytoskeleton assembly.	HB
GINS2	Over	Associated with DNA replication.	HS
HELLS	Over	Helicase involved in chromatin organization.	HS
HHIP	Under	Hedgehog-interacting protein, which is related to several developmental processes.	NA
HMMR	Over	Hyaluronic acid receptor, associated with metastasis formation.	HS
KIF11	Over	Kinesin family member, deeply related to spindle organization and mitotic progression.	HBS
KIF20A	Over	See KIF11.	HBS
KIF2C	Over	See KIF11.	HBS
KLF4	Under	Transcription factor associated with embryonic stem cell maintenance.	HBS
LIFR	Under	Cytokine receptor, which is heavily associated with the Leukemia Inhibitory Factor.	B
MAD2L1	Over	Member of the mitotic spindle assembling complex.	HBS
MCM6	Over	Required for DNA replication initiation through several processes.	HBS
METTL7A	Under	Putative methyltransferase.	NA
MT1E	Under	Metallothionein, which alters the intracellular concentration of heavy metals.	NA
MT2A	Under	See MT1E.	NA
NDRG2	Under	A hydrolase, which is related to Wnt-signaling.	NTR
NEK2	Over	Kinase that regulates several centrosome-associated events during mitosis.	HBS
PBK	Over	Kinase involved in MAPKK activation.	HBS
PLPP3	Under	Phospholipid phosphatase involved in the synthesis of glycerolipids.	NA
PRC1	Over	Involved in the mitotic spindle organization.	HBS
PRR11	Over	Related with cell cycle progression.	H
RCAN1	Under	Inhibitor of calcineurin A.	B
RRM2	Over	Subunit of a ribonucleotide reductase.	HBS
SELENOP	Under	Involved in selenium transportation.	NA
SOX4	Over	Transcription factor related to the regulation of embryonic development.	NA
TOP2A	Over	DNA topoisomerase.	HBS
TPX2	Over	Associated with microtubules spindle assembly.	HBS
UBE2C	Over	Member of the E2 ubiquitin-conjugating enzyme family.	HBS
ZBTB16	Under	Zinc-finger protein, associated to cell cycle progression.	HBS

*HBS, hub-bottleneck-switch; HB, hub-bottleneck; HS, hub-switch; H, hub; B, bottleneck; S, switch; NA, not applicable (This gene was not present in the network); NTR, no topological relevance*.

One primary observation that can be drawn from these results is that the tumoral process, in general, might be more closely related to the overexpression of conserved molecular machinery than to the underexpression of a given one. This is reflected not only based in the higher similarities found among the overexpressed genes but also in the higher percentage (70.2%) of overexpressed genes present in the most frequently expressed 47 DEG between all analyzed cancer types. Another unexpected result was that bladder cancer has the highest similarity to other cancers when it comes to underexpressed genes. In contrast, head/neck displayed higher similarity to lung cancer for overexpressed DEG. These results are in agreement with the heterogeneous nature of cancer but imply that some types are closer to others even if their similarity is non-intuitive. The 47 frequently expressed DEG identified were further investigated, as described in the next section.

Furthermore, the GSEs employed in the previous analysis were treated to specifically fit a machine learning protocol to be used as input in N3O (Grisci et al., 2019). N3O does not provide a DEG profile since this is a classification tool; however, it allows for the detection of the most representative genes in the tumoral process. By using a 10-fold validation protocol, we obtained a list of the most frequent features, taking into consideration only those present in at least three cancer types (Supplementary Table 19). N3O results point to genes of the same families as those identified by the DEG analysis, such as KIF4A, or close variants, like GINS1, as well for features that appeared as shared DEG among the cancer types, such as TOP2A. The top feature, ABCA8, appeared in five of 12 cancer types, being more prevalent in breast cancer. The overexpression of ABCA8 was recently reported to be a possible biomarker for breast cancer (Dvorak et al., 2017) and poor outcome in epithelial ovary cancer (Hedditch et al., 2014) in which ABCA8 was also listed as a feature. TOP2A, which is amongst our 47 most frequently expressed genes, and one of N3O's top feature, was also elected to have a possible prognostic value for lung cancer in a bioinformatic study using both the investigation of multiple GEO datasets, as well as PPI-networks (Ma et al., 2019).

This was not expected to be a feature that was common between all cancer types (a very few if any) or between the N3O findings and the classical DEG approach. N3O selected the features that are the most representative for a given cancer, not the vast majority, and the same feature appearing in 12 different types would thus be unlikely. In contrast, a feature that frequently appeared in more than one type could be a strong indication of major regulatory role. These relationships were further explored in the next section.

**Figure 3:**
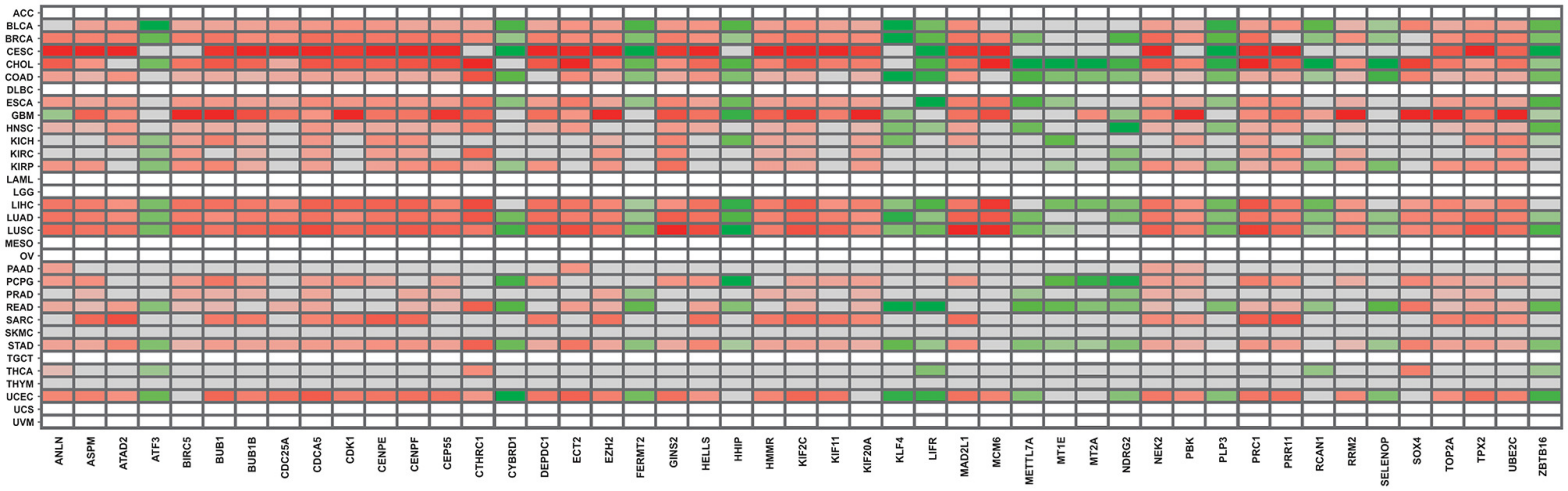
Gene expression panorama of the 47 frequently expressed genes, according to the DDBv3 database. The red-colored gradient indicates levels of higher expression, whereas the green-colored gradient indicates levels of lower expression.

Finally, to validate the results obtained from our approaches, we employed the DriverDBv3 database to match our results to TCGA gene expression data. We selected the same cancer types present in the overall survival analysis, as described in section 2. As can be observed in Figure 3, all gene expression results retrieved from the database are in accordance with our own, further validating our approach and results. Another recent study that analyzed different cancer datasets from GEO also observed some genes in common between our 47 DEG, such as ANLN, CDK1, ECT2, PRC1, NEK2, ASPM, RRM2, TOP2A, BUB1B, and CTHRC1 (Xue et al., 2020). Likewise, another pan-cancer study mentions a potential relevance for BIRC5, RRM2, and MCM6, present amidst our 47 DEG, among their findings (Cava et al., 2018).

### 3.3. Potential Regulatory Mechanisms

**Figure 4:**
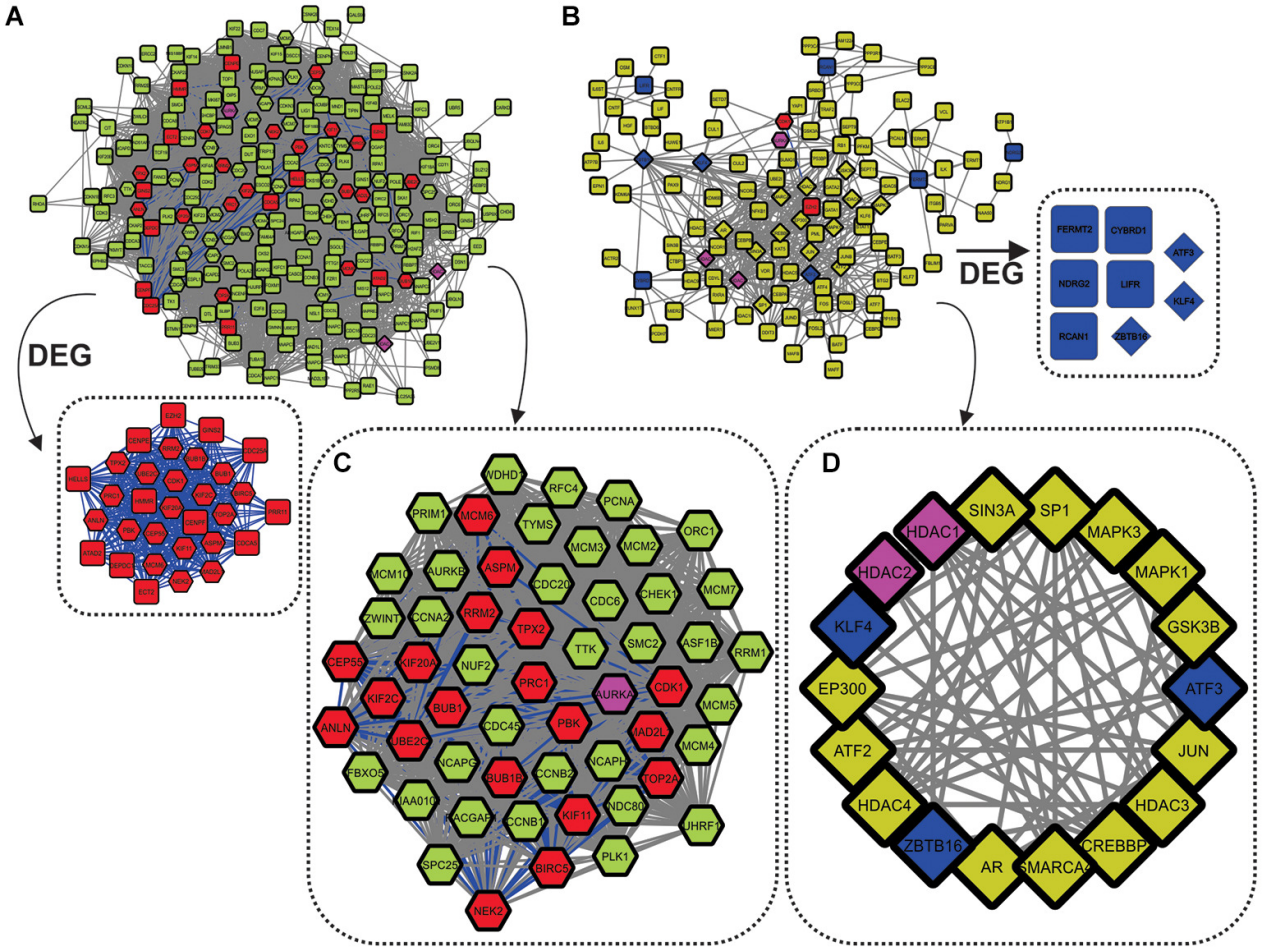
Networks built using the 47 DEG frequently expressed between all analyzed cancer types. **(A)** Over-DEG-Net. The red nodes depict the 31 (from 33) overexpressed genes frequently expressed between cancer types, whereas the green nodes show their first neighbors. The pink nodes are shared between the two networks. Over-DEG-Net is composed of 231 nodes and 9,144 edges, displaying high connectivity. **(B)** Under-DEG-Net. The blue nodes depict the 8 (from 14) underexpressed genes frequently expressed between cancer types, whereas the yellow nodes show their first neighbors. The network is composed of 115 nodes and 484 edges, displaying low connectivity. **(C)** The octagonal nodes refer to the Hubs-Bottlenecks-Switch (HBS) proteins from the overexpressed network. This subnetwork is composed of 55 nodes and 1,428 edges. **(D)** The diamond nodes indicate the Hubs-Bottlenecks-Switch (HBS) proteins from the underexpressed network. This subnetwork is composed of 18 nodes and 74 edges.

The most frequently expressed DEG was used as input to construct two separate PPI networks, one for overexpression and one for underexpression. We chose to expand the networks taking into consideration only the most preeminent connections. They were thus build using only the first shell proteins in the STRING database, and employing edges based on experiments and co-expression studies. This generated two topologically distinct networks (Figure 4). Some proteins did not connect to their respective networks, even after saturation tests on the first shell: (i) SOX4 and CTHRC1 for overexpressed genes and (ii) HHIP, MT1E, METTL7A, MT2A, SELENOP, and PLPP3 for underexpressed genes. We chose not to expand the networks more than the major first interactions because that usually overshadows the major interacting partners and the main associated biological processes. This resulted in two distinct networks, one for the overexpressed genes, named Over-DEG-Net (Figure 4A), and one for the underexpressed genes, named Under-DEG-Net (Figure 4B). Each PPI network was then analyzed to find the most topologically relevant nodes, which could be considered the top regulatory proteins. In this sense, the combination of the node degree, betweenness, and eigenvector centralities were used. Nodes with above-average node-degree scores are called Hubs; those with above-average betweenness scores are named Bottlenecks, and we named the ones with above-average eigenvector values “Switches”: nodes combining the three characteristics are thus referred to as HBS (Figures 4C,D), which possess regulatory roles within the cell (Scardoni et al., 2009).

Combining network topological studies using high-throughput data to understand diseases is already established as a useful approach to uncover its molecular complexity (Menche et al., 2015). In this sense, the structure of a biological network is the first evidence of how the underlying molecular mechanism is behaving, where genes that are mostly linked to a disease tend to form highly interconnected networks (Loscalzo and Barabasi, 2011). This tendency is observed for the Over-DEG-NET (Figure 4A), which comprises an incredibly connected network. In this sense, the 33 overexpressed genes used as input for the network construction were related to GOs deeply associated to all cancers, like a positive induction of cell cycle, deregulation of DNA repair, proteolysis, and kinase-related signaling pathways (Figure 2C and Supplementary Figure 1, Supplementary Table 16) (Hanahan and Weinberg, 2011; Pickup et al., 2014; Sanchez-Vega et al., 2018). The highlight of this result is that these genes were shared both microarray and RNA-seq analysis from 99 curated datasets in 14 cancer types, implying a possible frequently deregulated set of genes in tumoral tissues not observed so far, which is also in agreement with the observation made in the previous section. Even the HBS in the overexpressed genes formed a highly interconnected subnetwork (Figure 4C), which is in agreement with a conserved regulatory core.

We observed that there was a clear difference between the over and underexpressed genes shared between all analyzes in terms of biological function. This reflected on the network topology for the Under-DEG-Net (Figure 4B), which displayed a scarce and weakly interconnected network. This result suggests that the molecular machinery needed to drive the tumoral process is more closely related to the overexpression and deregulation of conserved cellular mechanisms than to highly specific ones. This observation was echoed in the HBS for the underexpressed genes, where little interaction between them was achieved, indicating that the tumoral process does not rely on the underegulation of core machinery but preferably on the overexpression of conserved mechanisms. The GO analysis was consistent with the expected for the tumoral process, with this network showing GO related to the negative regulation of angiogenesis and response to oxidative stress (Hanahan and Weinberg, 2011; Sharma et al., 2019) (Figure 2C and Supplementary Figure 1, Supplementary Table 17). In general, however, the GOs of Under-DEG-Net were not as informative as the one in Over-DEG-Net, which could be a reflection of its topological characteristics, as described previously.

The results obtained by N3O supports the previous conclusion. The top 20 features, which appeared in common between three or more cancer types, were used as input to create the PPI-Network (N3O-Net) (Supplementary Figure 3). Some targets did not show any connection to the network, even after the saturation test, and these targets were ABCA8, VEGFD, TRIM29, CA9, APOC1, FAP, CEACAM5, FOXF1, MFAP2, POGLUT2, and AGR2. Network topology was similar to the overexpressed genes network (Figure 4A), and 26 of the 33 overexpressed genes were part of the N3O-Net. N3O classifies the features which better represents a given class, in this case, the tumoral tissue. N3O-Net is thus based on significant classifiers of the tumoral tissues and their immediate neighbors. In agreement with the previous observation, N3O-Net depicts that the predominant drivers of the tumoral process are more related to a conserved core of overexpressed genes than to the underexpression of a given gene set since no underexpressed DEG was present in this network, furthering confirming N3O accuracy in identifying molecular markers.

N3O-Net was composed by a similar GO than the ones found for the Over-DEG-Net (Supplementary Figure 1, Supplementary Table 18). Another highlight of N3O-Net in terms of GO was that, in comparison to Over and Under-DEG-Net, it showed fewer artifacts, where most GO originally identified were submitted to the least amounts of manual filtering. This result echoes the nature of N3O results. Since N3O identifies genes that better classify a given condition, we can observe the most representative genes for the general tumoral process, such as cell cycle, DNA repair, chromatin modifications, and DNA metabolic processes, which explains why there are more bioprocesses in common with Over-DEG-Net than Under-DEG-Net.

Moreover, AURKA, which is one of the only three nodes in common between the Under and Over-DEG-Net, appeared in N3O-Net as a top feature. AURKA is deeply connected to the tumoral process, being involved in DNA repair, cell division, ATP production, and self-renewal of cancer stem cells (Li M. et al., 2018; Bertolin and Tramier, 2019). These results confirm the approach applied in this works and sustain the accuracy of the targets identified by the different bioinformatic approaches.

**Figure 5:**
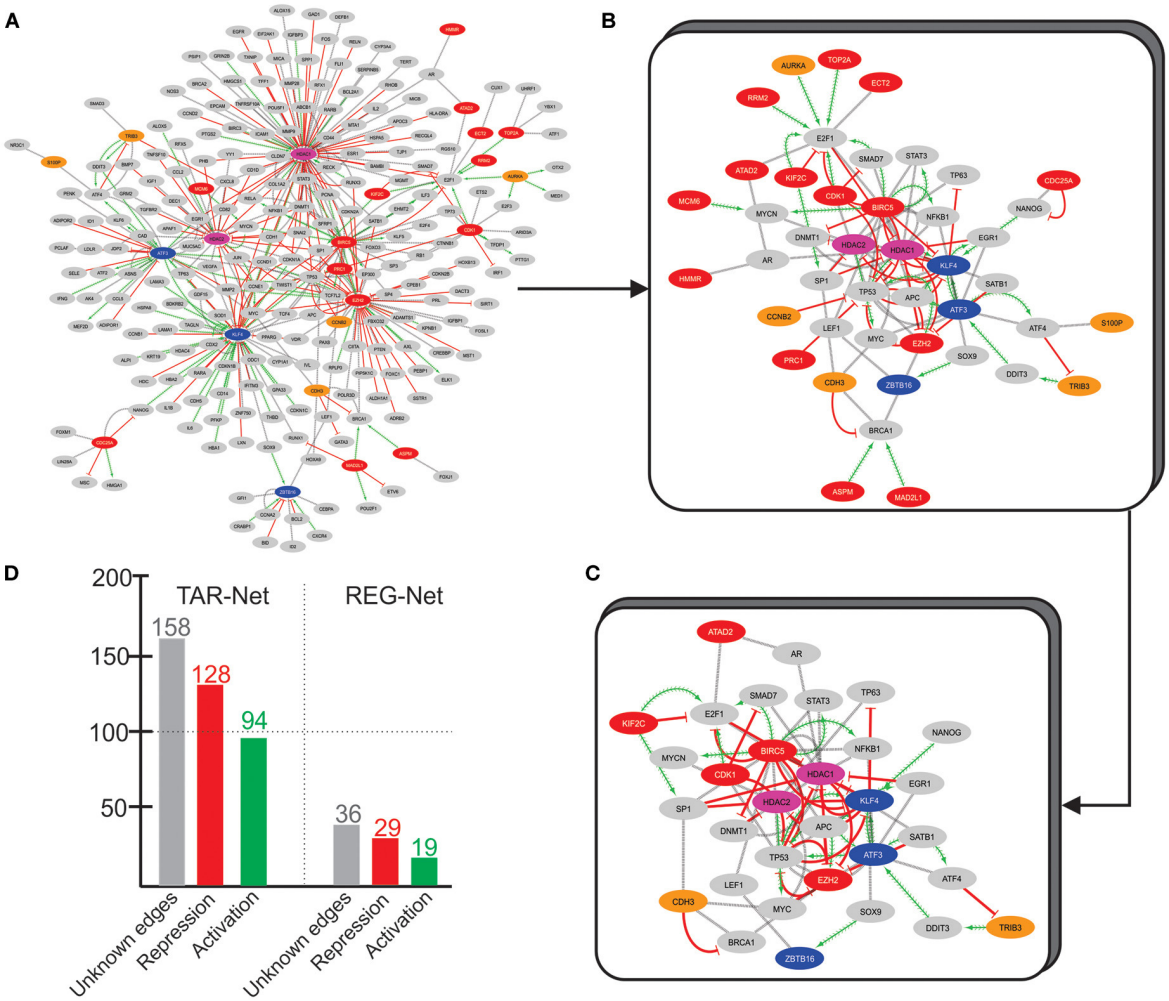
TAR-Net. **(A)** The initial network, composed by the DEG (Red and blue nodes, similar to the Over and Under-DEG-Nets), the nodes in common between the Over and Under-DEG-Net (pink nodes), N3O's top-features (orange nodes), and the transcription factors associated to them (gray nodes). The red edges represent repression, whereas the green edges depict activation. The gray edges are unknown connections. Unconnected nodes were excluded prior to the analysis. **(B)** REG-Net, composed only by the nodes predicted to be a regulatory network. This network is composed of 44 nodes and 97 edges. **(C)** Final subnetwork, containing only the transcription factors and DEG that have at least two other connection. The subnetwork thus contains 32 nodes and 84 edges. **(D)** Graph depicting the number of connections in TAR-Net and REG-Net.

To uncover more about this regulatory core, we used the 33 overexpressed genes from Over-DEG-Net, the 14 underexpressed genes from Under-DEG-Net, and the top-features identified by N3O (i.e., found in at least three cancer types, with at least 5 hits, see see Supplementary Table 19) as input seeds to prospect the transcription factors associated with them. This resulted in a new network, named Transcription-Associated Networks (TAR-Net) (Figure 5A), which was then analyzed to predict probable regulatory/signaling pathways within it. This new network was named Regulatory Network (REG-Net) (Figure 5B). REG-Net is composed of the genes predicted as part of the signaling network, the DEG, N3O's top-features, and the transcription factors associated with them. The interactions of TAR- and REG-Nets represents the type of regulation performed by the transcription factors. As it can be seen, not all DEG and N3O top features could be retrieved in the transcription factor prospection.

TAR-Net enabled the identification of major transcription factors that could regulate a possible core molecular pathway in the tumoral process, which was subsequently filtered to predict a potential regulatory core. The results of REG-Net sustain our previous findings since all underexpressed DEG classified as HBS appeared in this network, confirming their relevance in disease. The same happened for the overexpressed DEG, with the exception of EZH2, ECT2, CDC25A, HMMR, and ATAD2, which had lesser topological relevance.

We then focused on finding a new possible central mechanism that could influence the tumoral process based on REG-Net. Thus, we considered transcription factors as part of a “core regulatory pathway,” only those connected to at least two DEG, which resulted in a 32 node network (Figure 5C). Another interesting result is the nature of the regulation predominating in both TAR-Net and REG-Net. In this sense, aside from the unknown edges, repression appears to be the main regulation mechanism, followed by activation, which is the minority (Figure 5D).

These connections, however, were not exclusive for tumoral tissues, and due to the heterogeneous nature of cancer, the regulatory edges observed in this networks could not be preserved in the different tumoral types. Nevertheless, these connections provide an overview of the major type of regulation observed in these most frequently expressed DEG, as well as shows that there are numerous unknown connections that can still be explored in the context of cancer.

### 3.4. Overall Survival

The 47 DEGs identified to be frequently expressed, between all analyzed cancer types, were submitted to an overall survival analysis to investigate genes that were already associated with poor prognosis. The idea is to provide further discussion by selecting the top DEG that are already more frequently correlated to poor prognosis. The overall analysis is based on existing data, achieved by studying a different number of patients, from different genders, and distinct nationalities. It must thus be kept in mind that overall analysis results are subjected to these variables. Discrepancies, which are expected, are discussed at the end of the section. The overall analysis is not in any way a means of validation; it is but additional data.

**Figure 6:**
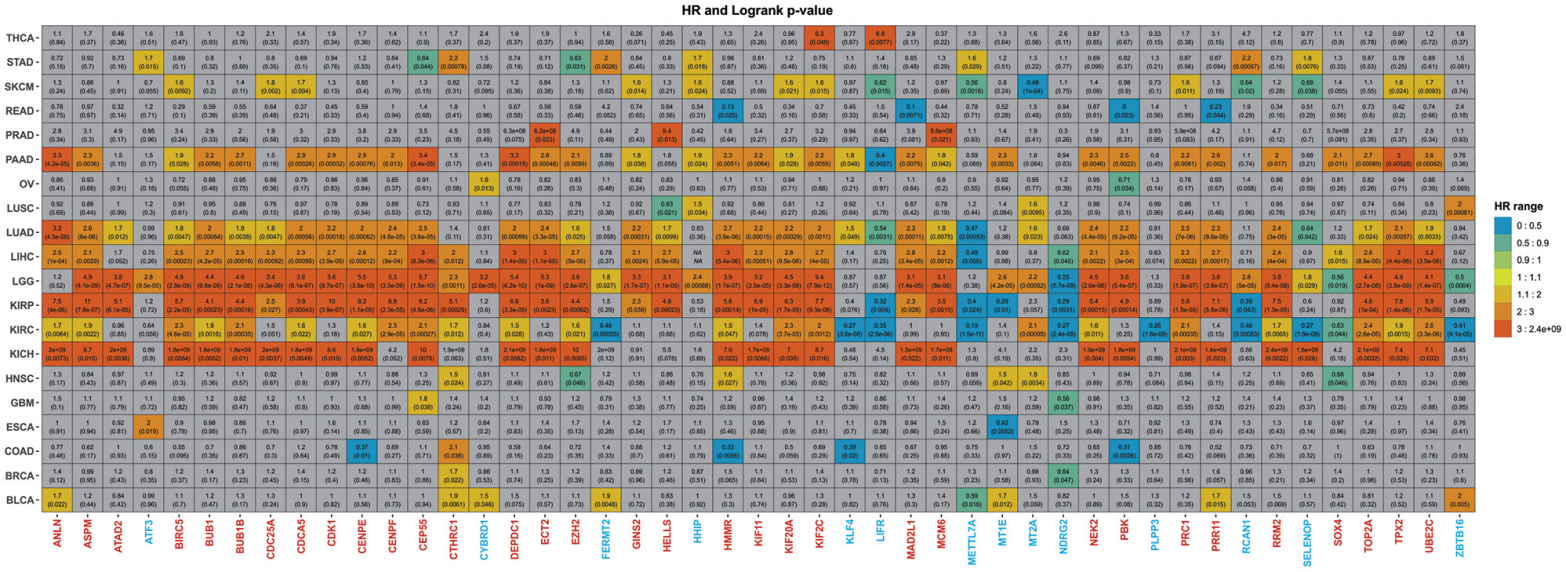
Summary of the overall analysis. The graph displays the 47 genes previously obtained. The gray squares represent genes that showed no statistical significance, whereas the colored ones presented significant p-values. The color scheme illustrates the HR score from lowest to highest. Underexpressed genes were colored blue, whereas overexpressed genes were painted red.

Among the 20 cancer subtypes in the overall analysis, LIHC ranks sixth in terms of incidence and fourth at cancer-related mortality (Siegel et al., 2019). In LIHC, 34 genes (32 overexpressed and two underexpressed) out of 47 genes were significantly associated with overall survival (Figure 6 and Supplementary Table 20) but not all had a relevant hazard ratio (HR). Even though a given could be associated with overall survival, it might not have a relevant HR. In this sense, we took into consideration HR ≥ 1 as an indicator of death risk (Sashegyi and Ferry, 2017). In accordance with our data, the 31 overexpressed genes also had a relevant HR and were related to poor survival, whereas the underexpressed genes (MTTL7A and NDRG2) did not.

Moreover, pancreatic cancer remains one of the neoplasias most difficult to treat with a survival rate of only 9% (Siegel et al., 2020) due to the lack of early symptoms presented in the metastatic stage (Adamska et al., 2017). In total, 33 (29 overexpressed and four underexpressed) from the 47 genes were significantly associated with overall survival (Figure 6 and Supplementary Table 20). Although the 29 overexpressed genes were related to poor survival in PAAD, three underexpressed genes (HHIP, KLF4, and MT1E) showed relevant HR.

It is interesting to note that all 29 genes present in PAAD were not only in common with LIHC, but 19 these genes, 65.5% (ANLN, ASPM, BIRC5, BUB1, BUB1B, CDK1, CEP55, KIF11, KIF20A, KIF2C, MAD2L1, MCM6, NEK2, PBK, PRC1, RRM2, TOP2A, TPX2, and UBE2C), were HBS in our network analysis (Table 1, Figure 4).

For the pan-kidney cohort (KICH, KIRC, and KIRP), Among the 47 identified genes, 18 genes (ANLN, ASPM, BIRC5, BUB1, BUB1B, CDCA5, CENPE, CEP55, DEPDC1, HMMR, KIF20A, KIF2C, NEK2, PRC1, RRM2, TOP2A, TPX2, and UBE2C) were in common between the three cancer types (Figure 6). The higher expression level of all these genes was associated with worse survival time and relevant HR, further validating our results. Among these genes 14 (77.7%), were HBS. For BLCA, seven genes showed relevant HR and were significantly associated with overall survival. Except for METTL7A, all underexpressed genes showed discrepancies with expected results (Figure 6).

Only one gene showed significance and relevant HR for GBM patients, CEP55. In contrast, 42 genes showed poor survival in LGG (Figure 6 and Supplementary Table 20). RRM2 is already recognized as a prognostic biomarker in glioma (Sun et al., 2019). There were some contradictions in LGG, however, as ATF3, CYBRD1, FERMT2, HHIP, MT1E, MT2A, RCAN1, and SELENOP showed relevant HR.

As for lung cancer, 36 genes were associated with overall survival for LUAD patients, where 33 had relevant HR (Figure 6 and Supplementary Table 20). Two discrepancies were observed: KLF4 and NDRG2. It is essential to highlight that a previous bioinformatics study also identified three of these genes (TOP2A, UBE2C, KIF20A) as hub genes that could be related to the prognosis of non-small cell lung cancer (Ni et al., 2018). In addition to the 36 aforementioned overexpressed genes in LUAD, only HELL, HHIP, MT2A, and ZBTB16 were associated with survival in LUSC patients (Supplementary Table 20).

Two genes showed both statistical significance and relevant HR in THCA, KIF2C, and LIFR, and four (CTHRC1, HMMR, MT1E, and MT2C) in HNSC (Figure 6 and Supplementary Table 20). LIFR, MT1E, and MT2C, which are underexpressed in our analysis, displayed relevant HR and statistical significance.

From the 18 genes related to overall survival in SKMC, FERMT2 was the principal contrariety. Still, elevated BIRC5, CDC25A, CDCA5, and UBE2C expression significantly predicted the increased hazard of dying from SKCM. In contrast, overexpression of METTL7A (HR = 0.56) and MT2A (HR = 0.48) increased survival—all in agreement with our results (Supplementary Table 20)—while in OV, CYBRD1 showed similar results. BRCA patients with high expression of CTHRC1 had shorter lifespans (HR = 1.7) however. Moreover, STAD, ESCA, COAD, and READ showed a mix of expected outcomes with discrepancies that followed a similar pattern that those discussed so far.

If we take into consideration the genes more frequently associated with poor survival that are also HBS, ANLN, ASPM, BIRC5, BUB1, BUB1B, CEP55, KIF20A, KIF2C, MCM6, NEK2, TPX2, and UBE2C appear to be the most prevalent for a worse outcome, which consistently supports previous results (Loscalzo and Barabasi, 2011). In summary, these results support our previous observations, where the overexpression of given molecular machinery is more likely to be responsible for driving the tumoral process than the underexpression of a given gene set. The discrepancies observed in the overall analysis also strengthen our observation that the underexpressed machinery is not the most conserved in the tumoral tissue.

## 4. Conclusion: How Do We Investigate Regulatory Mechanisms?

The pursuit to unravel molecular mechanisms that could be linked to the tumoral process is challenging not only due to the heterogeneous nature of the process but also thanks to the vast amount of conflicting information in the scientific literature. The same gene can be reported to have different functions in the same cancer type. For example, PAX2 (Al-Hujaily et al., 2015), Plexin 1 (Vivekanandhan and Mukhopadhyay, 2019), FOXC1 (Yang Z. et al., 2017), and PI3K (Thorpe et al., 2015) were all discussed to have divergent roles during the tumoral process.

It would consequently be implausible to assume that there is a global regulatory pathway to all cancer types, which will always be deregulated in every distinct tumoral tissue. We can, however, develop more accurate ways to assess possible core mechanisms, which could come close to this yet ungraspable reality. The most realistic approach, however, is to observe the frequency in which a molecular deregulation occurs. This reality is made possible due to the vast collection of expression datasets we have available. However, even though we have access to this vast collection of freely available studies, we still face a significant challenge: how should they be analyzed?

One of the first steps to take on this quest is to ensure data quality. It is a common misunderstanding, however, to assume that this can be achieved without dataset manual verification on several steps, especially when it comes to a disease such as cancer. In this work, we took various steps to not only ensure data quality but also created the most unbiased data pool we could achieve without compromising novelty and sampling. After analyzing 99 manually curated datasets derived from a rigorous filtering criteria by using a variety of integrative approaches, we obtained a potential regulatory pathway that could be associated with different cancer types. We detected 47 DEG that are frequently expressed between all cancer types analyzed in this work, where most genes were already associated to poor prognosis in different cancer types. Most DEG are classified as HBS in the constructed networks, which strengthens their role as drivers of a disease pathway. We also found a significant correlation between underexpressed genes in bladder cancer, where it achieved similarity with breast, colorectal, lung, and ovarian cancers. Additionally, head/neck cancer also had significant correlation between overexpressed genes to lung cancer, which is in agreement with previously published results (Hoadley et al., 2014; Amos et al., 2017).

Based on the gene expression, network, and overall survival results, it is likely that the tumoral process is more intimately associated with the overexpression of a frequently deregulated machinery. Although it is known that the underexpression of multiples genes is relevant to the tumoral process, it is less probable that they are central drivers or associated with poor prognosis in the short and long term. In this sense, we devised a molecular model of the most predominant targets for tumoral drivers.

Finally, although this work took the exhaustive effort to manually curate the GEO database, by accessing each study one-by-one, we understand that extra measures should be made to automate this process for the most reliable datasets. Even though not much can be done when it comes to analyzing experimental protocols to see if they fit any criteria, some aspects can be defined from scratch to ease manual labor. For example, platform choice is a relevant aspect that can significantly narrow down the initial search. In this work, we considered only the Affymetrix, Illumina, and Agilent manufactures because they are the major gene expression platforms. However, more filters could be added to narrow down the search, such as dismissing custom platforms or older versions of some choice platforms. Another aspect is to omit studies that lack the RAW format. Reanalyzing data should always be done from scratch to ensure the employed approaches' homogeneity and guarantee the data was accurately preprocessed.

Furthermore, another quick filter is to exclude works that lack at least three experimental and three WT samples. Numerous GEO studies do not provide the minimum number of samples for proper statistical analysis and can be eliminated from the start. Still, manual curation will always be a laboring process. The extent of the search is directly associated with the biological background's complexity and the study's primary goal.

## Data Availability Statement

The original contributions presented in the study are included in the article/Supplementary Material, further inquiries can be directed to the corresponding author/s.

## Author Contributions

BF was responsible for conceiving the work, data gathering, network and microarray analyses, overall survival analysis, manuscript writing, and critical discussion. JP was responsible for data gathering, microarray and RNA-seq analyses, overall survival analysis, manuscript writing, and critical discussion. IN was responsible for data gathering and gene expression data managing. SF was responsible for overall survival analysis and critical discussion. MD was responsible for project managing, data gathering, machine learning analysis, manuscript writing, and critical discussion.

## Conflict of Interest

The authors declare that the research was conducted in the absence of any commercial or financial relationships that could be construed as a potential conflict of interest.

## Abbreviations

B: bottleneck
BLCA: bladder carcinoma
BRCA: breast invasive carcinoma
COAD: colon adenocarcinoma
CuMiDa: curated microarray database
DEG: differentially expressed genes
ESCA: esophageal carcinoma
FS-NEAT: feature selection-neuroevolution of augmenting topologies
GBM: glioblastoma multiforme
GEO: gene expression omnibus
GO: gene ontology
GSE: GEO series
H: Hub
HB: hub-bottleneck
HR: hazard ratio
HS: hub-switch
HBS: hub-bottleneck-switch
HNSC: head-neck squamous cell carcinoma
KICH: chromophobe renal cancer
KIRC: kidney renal clear cell carcinoma
KIRP: kidney renal papillary cancer
LIHC: liver hepatocellular carcinoma
LGG: low grade glioma
LUAD: lung adenocarcinoma
LUSC: lung squamous cell carcinoma
ML: machine learning
OV: ovarian serous cystadenocarcinoma
PAAD: pancreatic adenocarcinoma
PPI: protein-protein interaction
PRAD: prostate adenocarcinoma
READ: rectum adenocarcinoma
REG-Net: regulatory network
S: switch
SKCM: skin cutaneous melanoma
STAD: stomach adenocarcinoma
TAR-Net: transcription-associated networks
TCGA: the cancer genome atlas
THCA: thyroid carcinoma.
