# Supplementary material for: Multi-Approach Bioinformatics Analysis of Curated Omics Data Provides a Gene Expression Panorama for Multiple Cancer Types
Source: Front Genet. 2020 Nov 23;11:586602. doi: 10.3389/fgene.2020.586602 (PMC7719697; doi:10.3389/fgene.2020.586602)
Supplement: Supplementary file 1 [file Data_Sheet_1.PDF]

[Supplementary Material]*Supplementary Material*

1 SUPPLEMENTARY DATA

1.1 Figures

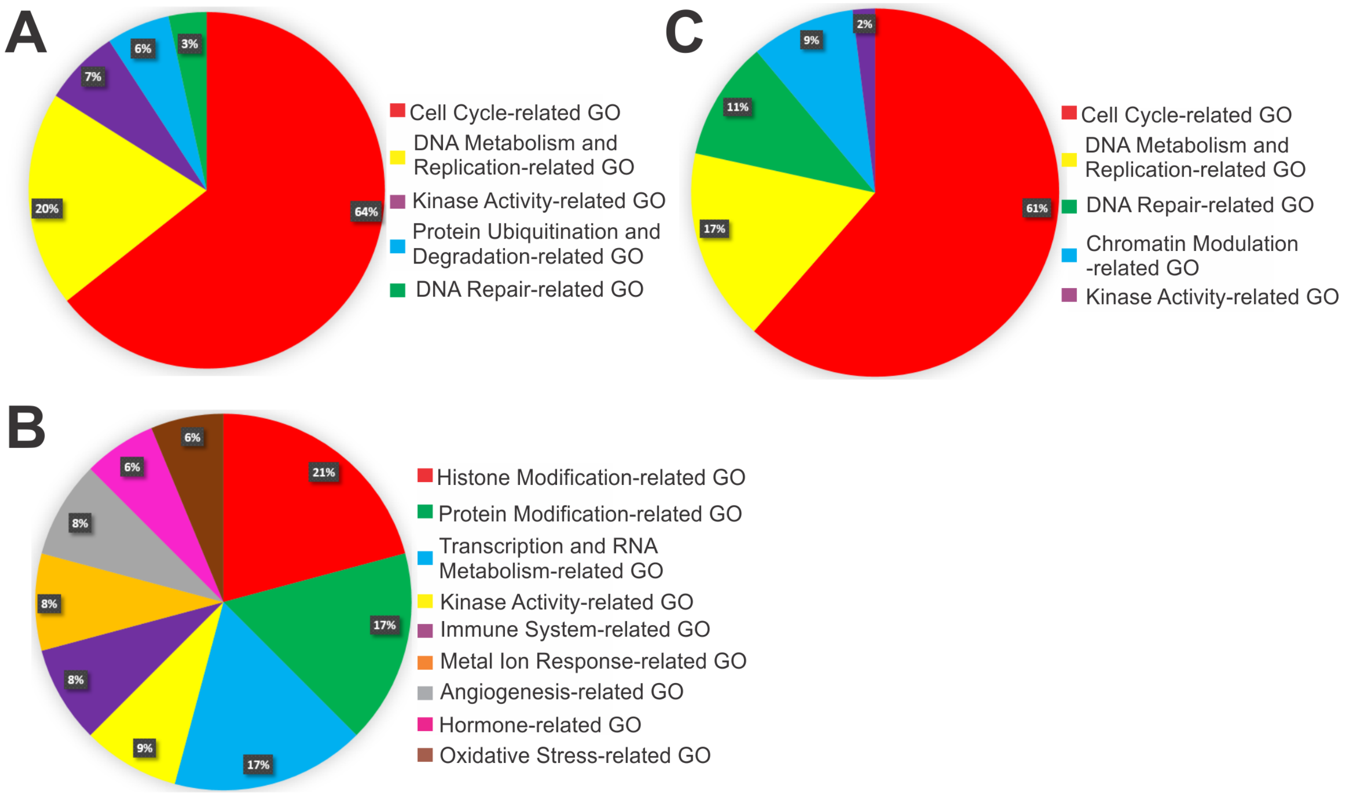

Figure S1. Gene Ontologies (GO) identified for the three networks. A) GO identified for Over-DEG-Net (**See main text and S-Table 18 in S-Material 2**). B) GO identified for Under-DEG-Net (**See main text and S-Table 19 in S-Material 2**) C) GO identified for N3O-Net (**See main text and S-Table 19 in S-Material 2**).

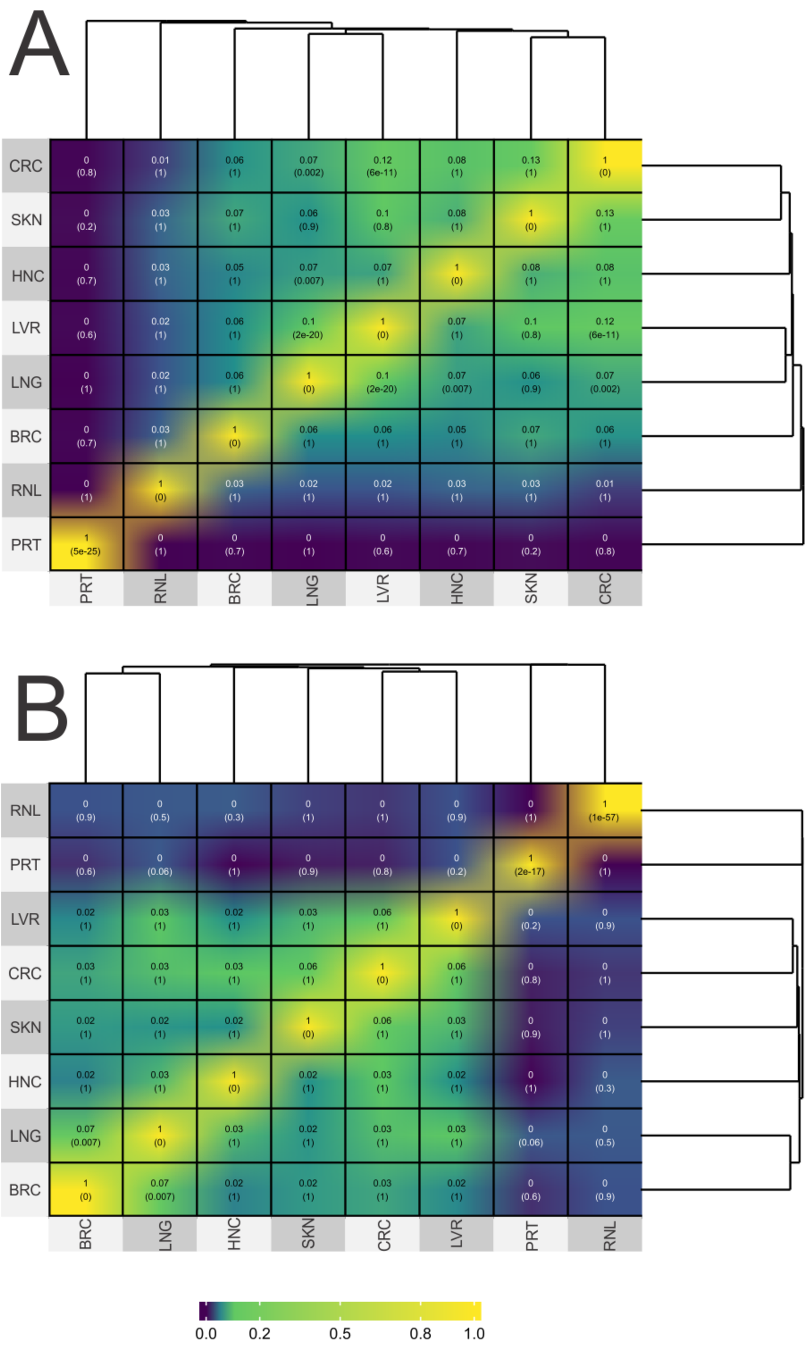

Figure S2. Jaccard index values for RNA-seq. A) Jaccard index matrix showing the similarity values for the overexpressed genes derived from the RNA-seq analysis. B) Jaccard index matrix showing the similarity values for the underexpressed genes derived from the RNA-seq analysis. White labels were added just for clarity. The values can also be seen in **S-Tables. XXX, see S-Material 2**. RNL = Renal [cancer]; PRT = prostate [cancer]; LVR = Liver [cancer]; CRC = Colorectal cancer; LNG = Lung [cancer]; BRC = Breast cancer; HNC = Head/Neck cancer; SKN = Skin [cancer].

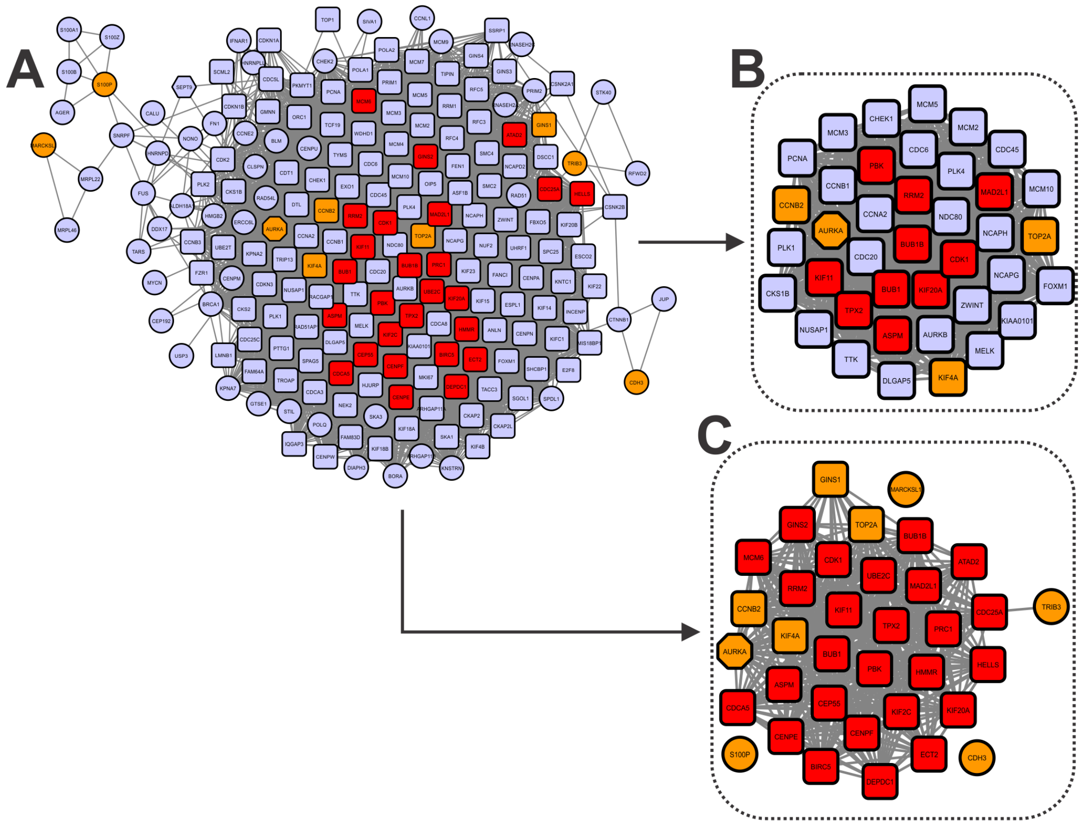

Figure S3. Network built using the top features identified by N3O from 12 cancer types and 4074 pooled samples. A) N3O-Net. The red nodes depicts the overexpressed genes in common between all caner types from the Over-DEG-Net, whereas the orange nodes represent the top identified features. The network is composed by 203 nodes and 8440 edges, displaying a high connectivity. B) The identified 39 HBS. C) Subnetwork composed only by the top features and the DEG from Over-DEG-Net.
